# dCLIP: a computational approach for comparative CLIP-seq analyses

**DOI:** 10.1186/gb-2014-15-1-r11

**Published:** 2014-01-07

**Authors:** Tao Wang, Yang Xie, Guanghua Xiao

**Affiliations:** 1 Quantitative Biomedical Research Center, University of Texas Southwestern Medical Center, 5323 Harry Hines Boulevard, Dallas, TX 75390, USA; 2 Harold C. Simmons Comprehensive Cancer Center, University of Texas Southwestern Medical Center, 5323 Harry Hines Boulevard, Dallas, TX 75390, USA

## Abstract

Although comparison of RNA-protein interaction profiles across different conditions has become increasingly important to understanding the function of RNA-binding proteins (RBPs), few computational approaches have been developed for quantitative comparison of CLIP-seq datasets. Here, we present an easy-to-use command line tool, dCLIP, for quantitative CLIP-seq comparative analysis. The two-stage method implemented in dCLIP, including a modified MA normalization method and a hidden Markov model, is shown to be able to effectively identify differential binding regions of RBPs in four CLIP-seq datasets, generated by HITS-CLIP, iCLIP and PAR-CLIP protocols. dCLIP is freely available at http://qbrc.swmed.edu/software/.

## Rationale

Eukaryotic genomes encode large numbers of RNA-binding proteins (RBPs), each of which has unique associating properties with RNAs and impacts the structure, localization, generation and function of both coding and non-coding RNAs [1,2]. Comparison of RNA-RBP interaction profiles across different conditions becomes increasingly important to understanding the function of RBPs and RNA regulation processes [3,4]. The advent of the crosslinking immunoprecipitation (CLIP) coupled with high-throughput sequencing (CLIP-seq) technique enables the investigation of RNA-RBP interactions at the genome level [5-7]. There are three versions of CLIP-seq experiments, high-throughput sequencing together with UV-crosslinking and immunoprecipitation (HITS-CLIP), photoactivatable-ribonucleoside-enhanced CLIP (PAR-CLIP) and individual-nucleotide resolution CLIP (iCLIP) [5-7], of which HITS-CLIP and PAR-CLIP are most commonly used. These two methods differ mainly by the crosslinking strategy being used. HITS-CLIP treats cells with UV light to crosslink proteins with RNAs and will introduce certain types of mutations in some of the CLIPed tags at crosslinking sites. For example, the mutations are specifically deletions if the crosslinked RBP is Argonaute (AGO) [8]. PAR-CLIP treats cells with photoreactive ribonucleotide analogs for incorporation into RNAs before UV treatment, which results in specific T → C or G → A substitutions depending on the type of nucleoside analog used [6]. One disadvantage of HITS-CLIP and PAR-CLIP is that reverse transcription must pass over the residual amino acids on the crosslink sites of RNAs. iCLIP overcomes this problem by employing a self-circularization strategy [9]. Also random barcodes are introduced to discriminate between PCR duplicates and unique cDNA products.

Although a few bioinformatics tools like PARalyzer, CLIPZ, wavClusteR and miRTarCLIP [10-13] have been developed to analyze a single CLIP-seq dataset, the quantitative comparison of multiple CLIP-seq datasets has only recently gained interest in the field [4,14,15]. Piranha [16] has been developed for CLIP-seq and Ribonucleoprotein immunoprecipitation followed by high-throughput sequencing (RIP-seq) [17] data analysis, and also provides a procedure for comparative analysis. However, the comparative analysis procedure in Piranha is relatively *ad hoc*, and does not utilize the spatial dependency among neighboring genomic locations, which is an important characteristic in creating differential binding profiles. A straightforward way to compare RNA-RBP interaction profiles across conditions is to analyze individual CLIP-seq data separately to identify the peaks (or binding sites) for each condition and then use coordinate overlapping or similar approaches to obtain common and differential binding sites. However, this *ad hoc* approach compares the results qualitatively but not quantitatively. For example, if a region is bound by an RBP under two conditions (for example, wild type versus knockout) with both significant enrichment but different binding intensities, the *ad hoc* approach will not be able to detect this region as a differential binding site. In addition, this *ad hoc* approach is over-sensitive to the cutoffs used for analyzing individual data, and has been shown to underestimate the similarity of two samples when applied to the analysis of multiple chromatin immunoprecipitation (ChIP)-seq experiments [18,19]. Therefore, a computational approach that can compare different CLIP-seq datasets simultaneously and quantitatively is needed.

The main challenge to quantitatively comparing genome-level sequencing profiles across conditions is that next-generation sequencing data usually contains relatively low signal-to-noise ratios [20,21]. Differences in background levels further complicate the analysis. To address these problems, several computational approaches have been developed for comparative ChIP-seq analysis, including ChIPDiff [22], ChIPnorm [23], MAnorm [24] and dPCA [25]. These computational approaches have greatly facilitated the understanding of dynamic changes of protein-DNA interactions across conditions. However, these computational approaches cannot be directly applied to CLIP-seq data to identify differential RNA-protein interactions, due to some inherent differences between ChIP-seq and CLIP-seq data. First, CLIP-seq data are strand-specific, while the tools designed for ChIP-seq experiments do not consider strands of peaks. Second, CLIP-seq experiments usually induce additional characteristic mutations in high-throughput sequencing reads, but the mutation information in the raw sequencing data is simply discarded in the bioinformatics software designed for ChIP-seq data analysis. Third, CLIP-seq reads are usually short, and the reads are not shifted or extended when counting tag intensities, but shifting or extension of reads is a necessary step in ChIP-seq analysis [26]. Fourth, CLIP-seq requires a much higher resolution (close to single nucleotide) in detection of RBP-binding sites, but ChIP-seq software usually work on a much lower level of resolution. For example, ChIPDiff is limited to 1 kb and ChIPnorm typically to a resolution of a few hundred base pairs. In addition, the method proposed by Bardet *et al.*[18] is not bundled as a portable software and takes about two days to finish. Therefore, we have developed the dCLIP software for detecting differential binding regions in comparing two CLIP-seq experiments.

dCLIP is a two-stage computational approach for comparative CLIP-seq analysis. As the first stage, a modified MA-plot approach was designed specifically to normalize CLIP-seq data across datasets to obtain high resolution results. As the second stage, a hidden Markov model (HMM) was developed to detect common or different RBP-binding regions across conditions. The HMM has a great advantage in modeling the dependency among adjacent genomic locations, which leads to improved performance in identifying differential binding sites. Here, we show that dCLIP can accurately identify RBP differential binding sites through the comparative analysis of four differential CLIP-seq datasets, including HITS-CLIP, PAR-CLIP and iCLIP experiments. In addition, we compared the performance of dCLIP and Piranha [16]. Our analysis shows that dCLIP can identify more biologically meaningful differential binding sites than Piranha.

## Availability

The source code and user manual for dCLIP are provided in Additional files 1 and 2 for documentary purpose, and are freely available at [27].

## Overview of the software

### Data preprocessing

**Figure 1:**
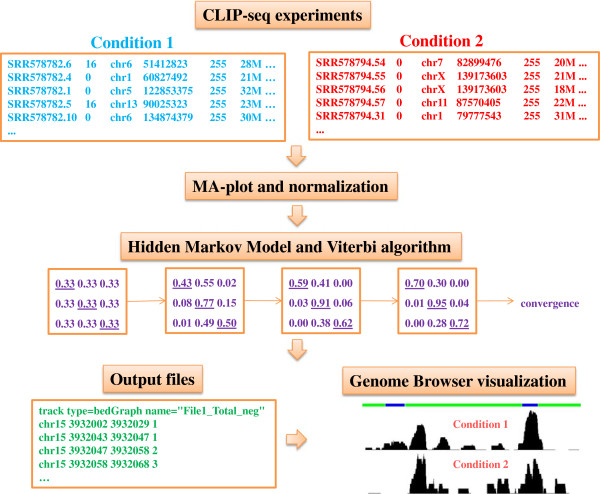
**Schematic representation of the dCLIP pipeline.** A summary of the major steps of dCLIP is provided as a flow chart. The format of the input and output files is also provided in the flow chart.

An overview of the dCLIP pipeline is shown in Figure 1. Data preprocessing is conducted in a strand-specific manner. For HITS-CLIP and PAR-CLIP, duplicate reads with the same mapping coordinates and the same strand are first collapsed to unique tags. The characteristic mutations are collected on all tags and written to separate output files. CLIP clusters are defined as contiguous regions of non-zero coverage in either condition and are identified by overlapping CLIP tags from both conditions. The tags that comprise each cluster retain their original condition identity. As a high resolution is needed for CLIP-seq analysis, dCLIP divides the clusters into bins of small length (the default is 5 bp) and calculates tag counts in each bin for both conditions. More specifically, the number of tags covering each base is calculated and the counts on all bases in each bin are summed to be the tag intensity count for that location. Therefore, the i-th bin in the j-th cluster has a pair of data points $x_{i}^{\left(j\right)}=\left(x_{i,1}^{\left(j\right)},x_{i,2}^{\left(j\right)}\right)$, where $x_{i,1}^{\left(j\right)}$ is the tag intensity count for the first condition and $x_{i,2}^{\left(j\right)}$ is the tag intensity count for the second condition.

iCLIP dataset preprocessing mainly follows that of Konig *et al.*[9], with minor modifications. Sequencing reads with the same random barcode represent PCR duplicates. Duplicates are removed and barcodes trimmed from the unique tags before mapping to the reference genome. A helper script, remove_barcode.pl, is provided in the dCLIP software to help users remove barcodes from Fastq sequencing files. After mapping, the first nucleotide upstream of each mapped cDNA, defined as the crosslink nucleotide, is expanded by a few nucleotides (specified by the users) in both downstream and upstream directions from its location, namely adding one to the tag counts on all bases in this short window. Therefore, the total tag count on each base is calculated as the sum of expanded cDNA counts covering that base and the mutant tag count will always be zero. Similarly, cDNA counts in both experimental conditions are summarized on the bin-level in regions of non-zero coverage.

### Data normalization

A normalization step is essential for an unbiased comparison because of the different sequencing depths of the two CLIP-seq samples. However, the common method of normalizing by total number of tags in high-throughput sequencing studies could be problematic, because of possibly different signal-to-noise ratios for different samples. We implemented the MA-plot normalization method, which was originally designed for normalizing microarray data [28] and later applied to ChIP-seq analysis [24]. When applying the MA-plot method to normalize microarray data, usually the expression value for each gene is used as a unit of normalization. When applying the MA-plot method to normalize multiple ChIP-seq data as in [24], read counts in the 1,000 bp windows centered on the summits of peaks are used as a data unit of normalization. However, in dCLIP, we modified the MA-plot method to normalize count data on the bin level, because high resolution is required in CLIP-seq data analysis. The $\left(M_{i}^{\left(j\right)},A_{i}^{\left(j\right)}\right)$ value of each bin is then defined as:

$$\begin{array}{l} M_{i}^{\left(j\right)}=\ln\left(x_{i,1}^{\left(j\right)}+c\right)-\ln(x_{i,2}^{\left(j\right)}+c)\\ A_{i}^{\left(j\right)}=\ln\left(x_{i,1}^{\left(j\right)}+c\right)+\ln(x_{i,2}^{\left(j\right)}+c) \end{array}$$

A small number c is added to each count value to avoid logarithm of zero count. We assumed that both conditions share a large number of common binding regions with similar binding strength. Therefore, a linear regression line *M* = *a* + *b* × *A* is fitted to bins whose $x_{i,1}^{\left(j\right)}$ and $x_{i,2}^{\left(j\right)}$ values are both larger than a user-defined cutoff. Because common binding sites should have similar binding strengths, the parameters derived from the regression model should capture the true scaling relationship between the two samples. This scaling relationship is extrapolated to the whole dataset, by subtracting a fitted M value from the linear regression model from the raw M value of every bin in all clusters. The adjusted M value is used in the following data analysis.

## Hidden Markov model

The HMM is a statistical Markov model in which the system being modeled is assumed to have spatial dependency between neighboring data units. RBP-RNA interactions involve a short stretch of RNA that can span up to a few bins [29]. This ensures the strong auto-correlation of tag counts in neighboring bins, which can be modeled by HMM. Therefore, we applied HMM to identify common and differential binding regions from the adjusted M values. As these adjusted M values come from many individual CLIP clusters, the HMM model has multiple observation sequences. During the statistical inference, all observation sequences share the same transition matrix and the same emission function.

The HMM has three possible states for each i-th bin in the j-th cluster:

$$\left\{\begin{array}{cc} I_{i}^{\left(j\right)}=0 & \mathrm{stronger}\;\mathrm{binding}\;\mathrm{in}\;\mathrm{condition}\;1\\ I_{i}^{\left(j\right)}=1 & \mathrm{non}‒\mathrm{differential}\;\mathrm{binding}\;\mathrm{site}\\ I_{i}^{\left(j\right)}=2 & \mathrm{stronger}\;\mathrm{binding}\;\mathrm{in}\;\mathrm{condition}\;2 \end{array}\right)$$

Accordingly, the transition matrix Π is a 3 × 3 matrix, whose element *π*_*r*,*s*_ is the transition probability $\mathrm{Pr}\left(I_{i}^{\left(j\right)}=\left(s\right|I_{i-1}^{\left(j\right)}=r\right)$ Given state $I_{i}^{\left(j\right)}$, the adjusted M values are fitted by a three-component normal mixture model. Because the common peaks that are determined by similar mechanisms in both conditions are normalized towards the same binding strength, the middle normal component is assigned a mean of zero. To avoid unreasonable assignment of bins to hidden states when the adjusted M values are extremely large or small, the three normal components are all assumed to have the same variance. Also, to simplify the problem, the means of first and third normal components are assumed to have the same absolute value but different signs.

To estimate the parameters for the HMM, we adopted an empirical-based method by fitting the adjusted M values to a three-component Gaussian mixture model.

$$\begin{array}{l} f\left(M_{i}^{\left(j\right)}|\sigma ,\mu ,p\right)=p\;\times \;\frac{1}{\sqrt{2\pi }\sigma }\;\times \;e^{\frac{{\left(M_{i}^{\left(j\right)}+\mu \right)}^{2}}{2\sigma^{2}}}+\left(1-2p\right)\;\\ \;\times \;\frac{1}{\sqrt{2\pi }\sigma }\;\times \;e^{\frac{{\left(M_{i}^{\left(j\right)}\right)}^{2}}{2\sigma^{2}}}+p\;\times \;\frac{1}{\sqrt{2\pi }\sigma }\;\\ \;\times \;e^{\frac{{\left(M_{i}^{\left(j\right)}-\mu \right)}^{2}}{2\sigma^{2}}} \end{array}$$

Since we assume that most sites would not show changes in their binding between conditions, the second component should dominate the mixture distribution. The first and third components can be treated as outliers if we solely focus on the second component. We then apply a median absolute deviation method [30] to robustly estimate the standard deviation to estimate *σ*, by equating $\hat{\sigma }=\mathrm{median}\left(\left|M‒\mathrm{median}\left(M\right)\right|\right)\times 1.4826$.

The other parameters *P* and *μ* are estimated by a recombinant method that combines method of moments estimator and maximum likelihood estimator [31]. Simply speaking, the second moment and sample second moment of the mixture distribution are given by:

$$\begin{array}{l} \mu_{2}=p\times \left(\mu^{2}+{\hat{\sigma }}^{2}\right)+\left(1-2p\right)\times {\overset{⌢}{\sigma }}^{2}+p\times \left(\mu^{2}+{\overset{⌢}{\sigma }}^{2}\right)\\ {\hat{\mu }}_{2}=\frac{\sum {\left(M_{i}^{\left(j\right)}\right)}^{2}}{n} \end{array}$$

By equating the above two formulas, we could get a constraining relationship between *P* and *μ*. The likelihood function was written as:

$$\begin{array}{l} L\left(p,\mu |M_{i}^{\left(j\right)},\hat{\sigma }\right)\\ =\prod_{i,j}f\left(M_{i}^{\left(j\right)}|\hat{\sigma },\mu ,p\right)\\ =\prod_{i,j}\left(p\times \frac{1}{\sqrt{2\pi }\hat{\sigma }}\times e^{\frac{{\left(M_{i}^{\left(j\right)}+\mu \right)}^{2}}{2{\hat{\sigma }}^{2}}}+\left(1-2p\right)\right)\left(\times \frac{1}{\sqrt{2\pi }\hat{\sigma }}\times e^{\frac{{\left(M_{i}^{\left(j\right)}\right)}^{2}}{2{\hat{\sigma }}^{2}}}+p\times \frac{1}{\sqrt{2\pi }\hat{\sigma }}\times e^{\frac{{\left(M_{i}^{\left(j\right)}-\mu \right)}^{2}}{2{\hat{\sigma }}^{2}}}\right) \end{array}$$

So, using grid approximation, we obtain a pair of $\hat{p}$ and $\hat{\mu }$ that maximize the likelihood function and also maintain the constraint at the same time.

The emission probabilities are calculated from the fitted model and fixed for each bin in different states before the iterations of HMM start. To find the chain of most likely hidden states, given the observations and the model, a Viterbi dynamic-programming algorithm is employed to infer the hidden state $I_{i}^{\left(j\right)}$.

### Data visualization

Finally, adjacent bins inferred to be in the same state are concatenated into continuous regions. A BED file is then generated to be uploaded to the University of California Santa Cruz (UCSC) Genome Browser, each entry of which is one continuous region in the same state. In addition, a TXT file is generated that describes the inference results of each bin in more detail. Eight bedGraph files are generated that store the total or mutant tag counts for both conditions and both strands. These files can also be directly uploaded to the UCSC Genome Browser for visualization. Examples of output files from the dCLIP pipeline are provided in Additional file 3.

## Implementation

The dCLIP software was implemented in the Perl programming language. Perl (versions above 5.16) together with two Perl modules PDL and PDL::Stats are needed to run the program. The implementation is supported on all major operating platforms.

The dCLIP software inputs SAM format alignment files of the two conditions to be compared. The SAM format files can be in single-end mode or paired-end mode. The users can specify parameters such as bin size, minimal number of tags in a cluster, the number of nucleotides to expand for cDNA counts (iCLIP), the type of characteristic mutations to be profiled and the stop conditions for the HMM.

## Case studies

### miR-155/AGO HITS-CLIP dataset

We used dCLIP to analyze the miR-155/AGO HITS-CLIP dataset from Loeb *et al.*[4], where the authors were interested in revealing miR-155-dependent AGO protein-binding sites. During microRNA (miRNA) biogenesis, double miRNAs are incorporated into the RNA-induced silencing complex [32] after being processed by Dicer. The miRNA/miRNA* duplex is then separated within the AGO protein and only one strand (the ‘guide strand’) will be retained before binding to mRNA targets. As a result, AGO protein, as one of the key catalytic components of the RNA-induced silencing complex, serves as a scaffold for miRNA and mRNA interaction. In this study [4], miR-155 knockout mice were generated, and CD4+ T cells were extracted from both the wild-type mice and the miR-155-knockout mice for performing HITS-CLIP experiments. Therefore, the differential AGO protein-binding sites should provide important cues for miR-155 targeting events.

**Figure 2:**
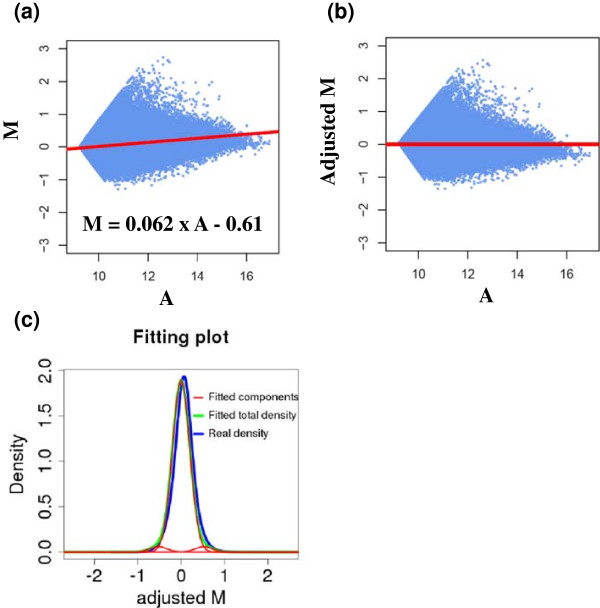
**MA-plot followed by linear regression. (a)** The MA plot of all the bin count data before normalization. **(b)** The MA plot of all the bin count data after normalization. The adjusted M value is the raw M value at each data point minus the fitted value from the regression line. **(c)** The three-component normal mixture model fitted to the adjusted M values after normalization. The blue line shows the real density. The green line shows the fitted density, which is the sum of the three individual components shown as red lines.

Raw sequencing reads were downloaded from [GEO:GSE41288] and mapped to the mm9 reference genome by Bowtie [33]. Unmapped reads were aligned by Novoalign (Novocraft Technology, Selangor, Malaysia). There were a total of 37 million mapped reads for the wild-type condition, and 34 million mapped reads for the miR-155 knockout mouse. A total of 58,872 individual clusters were identified and divided into a total of 1,131,870 bins. The adjusted M values had an autocorrelation of 0.81, corroborating the feasibility of using HMM for identifying common and differential binding sites for CLIP-seq datasets. For this dataset, the majority of the AGO binding sites that represent potential target sites of other miRNAs should remain overall unchanged after miR-155 knockout, as miR-155 knock out only directly influences a small proportion of AGO binding sites, thus satisfying the underlying assumption of the dCLIP algorithm as described above. dCLIP conducted MA-plot followed by linear regression to normalize the two CLIP-seq samples (Figure 2a,b), and fitted a three-component mixture model to the adjusted M values (Figure 2c). After HMM had reached convergence, the updated Π matrix showed that the HMM had probabilities of 0.76, 0.97 and 0.79 for the next bin to be in the same state as the previous bin, for state 0, state 1 and state 2, respectively. This confirmed again the assumption of strong dependencies between neighboring bins.

**Figure 3:**
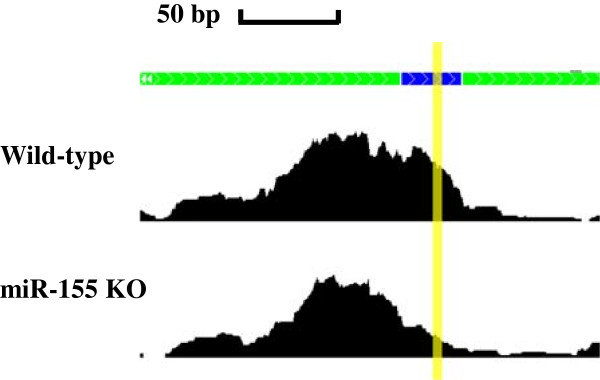
**An example of putative miR-155 target sites identified by dCLIP.** The tag intensities in the wild-type and miR-155 knockout conditions are shown. Green bars indicate regions with the same binding strength, and blue bars indicate regions with stronger AGO binding in the wild-type than the knockout condition. The yellow rectangle indicates the 6-mer seed motif of miR-155. KO, knockout.

**Figure 4:**
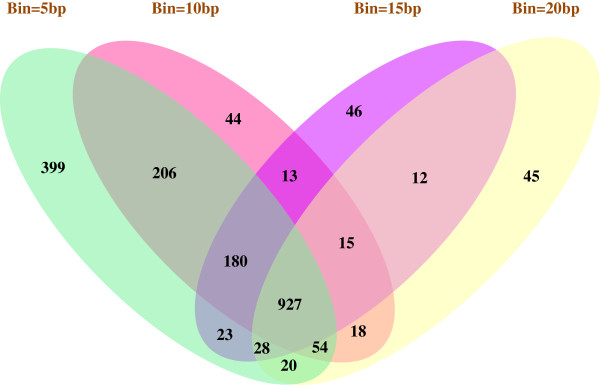
**Sensitivity analysis of the bin size parameter.** The four-set Venn diagram shows the overlap of the genomic regions that have stronger binding in the wild-type mouse than the miR-155 knockout mouse and have an average tag intensity of at least 30 in the wild-type condition, found by using different bin size parameters.

Using dCLIP, we identified 77,589 regions with no differential binding, 7,594 regions with stronger binding in the miR-155 knockout condition and 19,306 regions with stronger binding in the wild-type condition. The number of regions with stronger binding in wild-type was much larger than the number of regions with stronger binding in miR-155 knockout, which was reasonable because diminishing of AGO protein binding at miR-155 target sites should be the main effect of miR-155 knockout. To narrow down the list of sites for analysis, 1,469 regions that had stronger binding and an average tag intensity of at least 30 in the wild-type condition were selected. Figure 3 shows an example target region located in the 3’ untranslated region (UTR) of the *Zfp652* gene. A bin size of 10 bp was chosen for this analysis and sensitivity profiling across a big range of bin size values showed that the majority of these 1,469 regions were constantly detected regardless of the bin size used (Figure 4).

**Figure 5:**
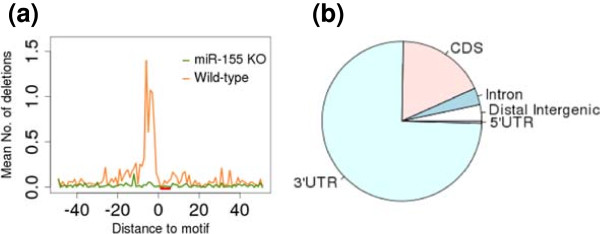
**Counts of nearby deletions and genomic annotations of the 150 binding sites identified by dCLIP. (a)** Deletion mutations around miR-155 seed motif matches. The x-axis is the relative distance to the miR-155 seed motif match, and the y-axis is the mean number of deletions per putative target site. The red rectangle shows the position of the miR-155 motif. **(b)** Overlap of the 150 AGO protein binding sites and RefSeq genes. Distal intergenic refers to the genomic regions that are not coding sequences, 3ʹUTRs, 5ʹUTRs or introns. CDS, coding sequences; UTR, untranslated region.

Among the 1,469 genomic regions, 150 regions contained at least one 6-mer seed motif of miR-155 (GCATTA). These represented the putative miR-155 targets, as evidenced by the accumulation of a large number of deletion mutations immediately upstream of the miR-155 seed motif matches in the mapped reads (Figure 5a). Among these 150 regions, 114 overlapped with the 3ʹUTR of at least one gene (Figure 5b), consistent with previous knowledge of the miRNA targeting mechanism. In the original publication [4], by using an *ad hoc* approach, the authors identified a list of 108 targets that satisfied the same criteria: stronger binding in wild-type than in knockout; located in the 3’UTR of at least one gene; and at least one seed motif match. There were 57 common binding sites shared by the 114 sites found by dCLIP and 108 sites found by the *ad hoc* approach. Although dCLIP identified more binding regions containing the seed motif of miR-155 than the original *ad hoc* approach, the total number was still relatively small. We believe the main reason for this was due to the non-canonical seed match. Of the 1,469 genomic sites identified by dCLIP, we searched for seed-like motifs with one mismatch (for example, GCACTA) or one bulge (for example, GACATTA) to the perfect 6-mer seed miR-155 motif (GCATTA), and found a total of 58 seed-like motifs with one bulge and 441 seed-like motifs with one mismatch, as well as 150 motifs having perfect matches. Although not all of, and not only, these sites are non-canonical miR-155 target sites, the numbers indicate the prevalence of possible non-canonical binding sites for miR-155.

**Figure 6:**
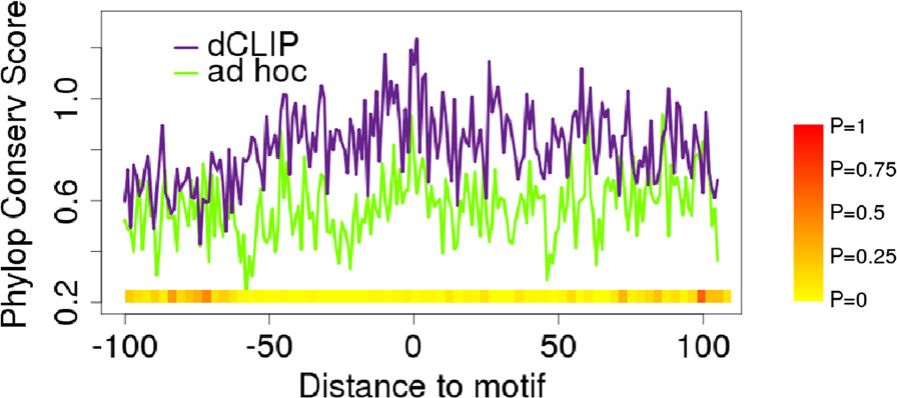
**Conservation scores of AGO binding sites found by only one method and not the other.** The y-axis is the phyloP conservation scores, and the x-axis is the relative distance to the start of miRNA seed match. The purple and green lines show the averaged conservation scores for the dCLIP-specific and *ad hoc* method-specific sites. The color bars at the bottom show the *P-*values of one-way t tests of the conservation scores in a 3 bp moving window between the *ad hoc* and dCLIP methods.

To assess the reliability of the inference results from the dCLIP software, we studied the conservation scores and gene expression levels of the targets identified by only one method and not the other. We fetched the phyloP (phylogenetic p-score) conservation scores in a 200 bp window covering the seed motif matches of miR-155. Then the conservation scores were averaged for the 57 sites found only by dCLIP and the 51 sites found only by the *ad hoc* method. The sites found only by dCLIP had much higher average conservation scores around the miRNA seed matches than those identified only by the *ad hoc* method (Figure 6). Because functional miRNA binding sites tend to be conserved across species, the results indicate that dCLIP identified more reliable differential binding sites than the *ad hoc* approach. One interesting thing to note is that while most studies focus only on the degree of conservation within seed motif matches [34,35], our results seem to suggest that miRNA targets are located in broader contiguous regions conserved across multiple species.

**Figure 7:**
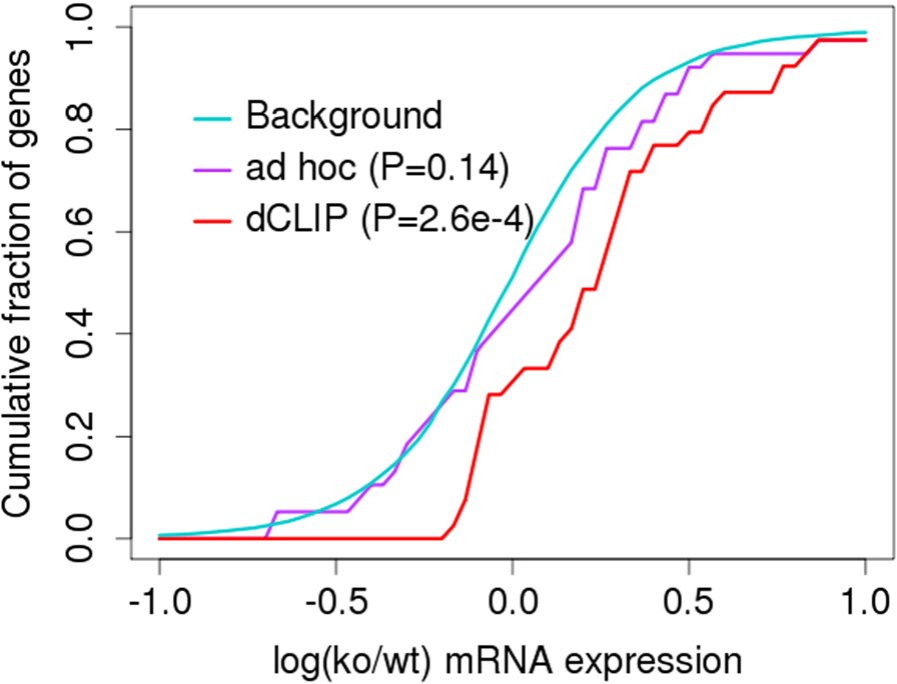
**The expressional differences of target genes found by only one method and not the other.** The expression profiles of all genes constitute the background distribution. *P*-values were calculated by a one-sided Kolmogorov-Smirnov test comparing method-specific genes with the background distribution. The x- axis shows the cutoff, and the y- axis shows the percentage of genes that have differential expression greater than the cutoff (as compared to background). ko, knockout; wt, wild-type.

miRNAs have been shown to suppress gene expression through translational repression and mRNA decay [36-38]. Therefore, we expected that miR-155 target genes would be mainly upregulated after miR-155 knock out, and that these changes could be measured at the mRNA level. We identified genes whose 3’UTRs had at least one putative miR-155 target site, and used the microarray experiment data from the original publication to calculate the expressional changes after miR-155 knockout. We found that dCLIP-specific target genes showed significant upregulation after knockout compared to the background distribution, whereas the *ad hoc*-specific target genes did not (Figure 7). Therefore, the gene expression results also confirmed that dCLIP outperforms the *ad hoc* method in identifying reliable differential AGO binding sites.

### *FMR1* PAR-CLIP dataset

To show that dCLIP can also handle PAR-CLIP datasets, we applied the dCLIP software to a PAR-CLIP dataset where the RBP under investigation is fragile X mental retardation protein (FMRP) [39]. The *FMR1* RBP family comprises three members, *FMR1*, *FXR1* and *FXR2. FMR1* encodes for many isoforms, of which isoform 7 is predominantly expressed [40]. The authors identified two major binding motifs of FMR1, ACTT/ACTG and AGGA/TGGA. The authors generated a recombinant FMR1 isoform 7 protein with a point mutation I304N in the KH2 domain. Through electromobility shift assays and PAR-CLIP experiments conducted with the wild-type and I304N proteins, the authors found the KH2 domain to be specific for binding to the ACTT/ACTG motif. Therefore, diminished binding to the ACTT/ACTG motif, rather than the AGGA/TGGA motif, should be the primary effect of the point mutation.

**Figure 8:**
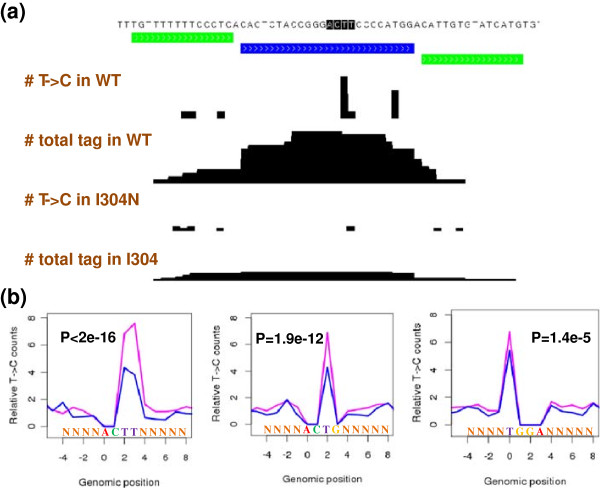
**The analysis of the *FMR1 *dataset by dCLIP. (a)** An example of FMR1 binding site with stronger binding in the wild-type condition than the I304N condition. The total tag counts and T → C mutant tag counts are shown. Green bars indicate common binding regions, and blue bars indicate regions with stronger binding in the wild-type than the I304N condition. The peak heights are scaled proportionally to the total sequencing depths of the two samples. **(b)** The relative counts of T → C mutations on top of all ACTT/ACTG and TGGA motifs found within the 9,859 binding sites. The T → C mutation counts on the T bases in these motifs are divided by the total T → C counts in a 30 bp window as background distribution. Because the sequences surrounding these motifs vary and for each base outside these motifs only a fraction of the 9,859 binding sites have T base, they are all marked as N. The *P*- values shown are for testing the differences in the proportions of T → C counts on top of each motif out of the total T → C counts in the 30 bp window between the wild-type condition (pink line) and I304N condition (blue line). WT, wild-type.

We downloaded the raw sequencing files from [GEO:GSE39686]. Adapters were trimmed and the sequencing reads were aligned to the hg19 genome using Bowtie [33]. Then we analyzed the mapping files with the dCLIP software. dCLIP found a total of 9,859 FMR1 isoform 7 binding sites that had stronger binding strength in the wild-type than in the I304N mutant condition and had at least an average tag intensity of three in the wild-type condition. We show one such binding site in Figure 8a. This binding site locates in the 3’UTR of the *Smad4* gene. The blue bar marks the binding region that has reduced binding upon mutation. Both the total tag counts and T → C mutation counts are shown.

We further calculated the number of T → C mutations that occur on top of all ACTT, ACTG and TGGA motifs found within those 9,859 binding sites in both the wild-type and I304N condition (Figure 8b). The T → C mutation counts on the T bases in these motifs were divided by the total T → C counts in a 30 bp window as the background distribution. Because the AGGA motif does not have a T base, there were no T → C mutations on top of this motif and this motif was thus not included in this analysis. The normalized number of T → C mutations in the I304N condition was smaller than the number of T → C mutations in the wild-type condition for the ACTT/ACTG motif as well as the TGGA motif, consistent with these sites having weaker binding in the I304N condition. The extent by which the relative T → C mutation counts decreased in the I304N condition was much more significant for the ACTT/ACTG motif (*P* <2e^-16^ for ACTT, *P* = 1.9e^-12^ for ACTG) than the TGGA motif (*P* = 1.4e^-5^). This was expected because the I304N point mutation locates in the KH2 domain responsible for binding to the ACTT/ACTG motif. Because the ACTT/ACTG and TGGA/AGGA motifs always occur in adjacent or nearby regions on the genomic sequence, a loss of binding affinity to the ACTT/ACTG motifs by the I304N mutation should lead to a secondary, weaker effect on the binding of the protein to neighboring TGGA/AGGA motifs. Overall, the analysis of this *FMR1* PAR-CLIP dataset shows that dCLIP also performs well on PAR-CLIP datasets.

### miR-124/AGO HITS-CLIP dataset

We also benchmarked the performance of dCLIP against Piranha [16], which provides a procedure for comparative CLIP-seq analysis. In the Piranha software, read intensities are first counted and binned. It also defines a set of properties that vary along with the count data. These one or more properties could be either count or other types of data. For example, one property could be the binned count data of the second condition, which enables Piranha to identify differentially regulated RBP binding sites in this scenario. The count of the second condition is used to scale the count of the first condition and the scaled count data are used to fit a model. For fitting the statistical model, a variety of options are provided, including Poisson Model, Negative Binomial Model, Zero Truncated Poisson Model and Zero Truncated Negative Binomial Model. Finally, bins with significant *P*-values are identified as differential binding sites.

We compared the performance of the dCLIP and Piranha software on the miR-124/AGO HITS-CLIP dataset produced from the original publication of Piranha [16]. In this dataset, HEK293 cells were transfected with miR-124 to identify its targets by comparison against non-transfected cells. Because miR-124 is not endogenously expressed, the AGO binding sites that are enriched in the transfected condition compared with the non-transfected condition should mostly mark miR-124 binding sites. We downloaded the raw sequencing data from SRA056343, trimmed adapters and then aligned the Fastq files to the hg19 genome using Bowtie [33] and Novoalign. Then dCLIP and Piranha (using the Poisson model) were used to identify the differential AGO binding sites enriched in the transfected condition. For both tools, a bin size of 5 bp was used.

**Figure 9:**
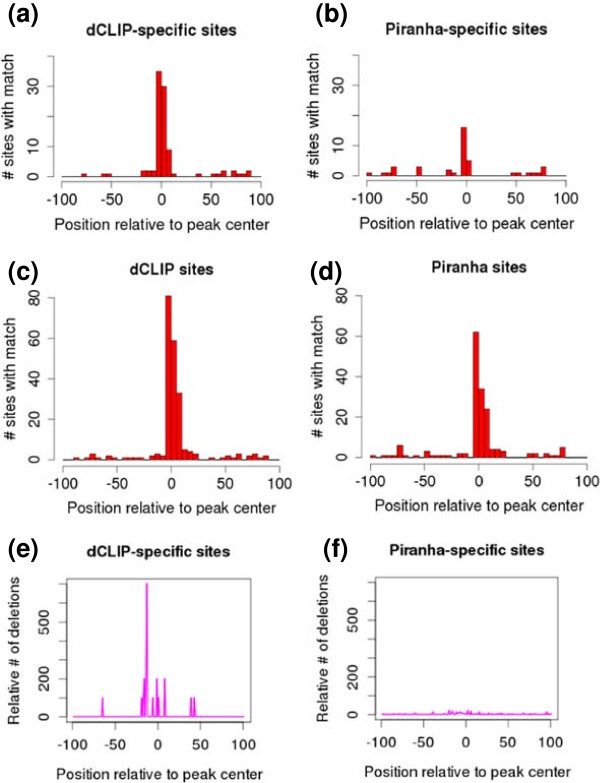
**Comparison of the dCLIP software and the Piranha software. (a,b)** Motif match counts within target sites found by only one method and not the other. Targets sites were extended to 100 bp both upstream and downstream from the peak center. Then the RNA sequences covered by the target sites were scanned for matches to any 7-mer from the reverse-complement of the mature miR-124 sequence (GGCAUUCACCGCGUGCCUUA). The x-axis is the relative distance of motifs to the peak centers and the y-axis is the number of sites with motif matches. **(c,d)** Total motif match counts within target sites found by each method. **(e,f)** Targets sites were extended to 100 bp both upstream and downstream from the peak center. Then the deletion mutations were counted within the method-specific target sites in both miR-124 transfected and the control conditions. The mutation count number in the transfected condition was divided by the mean count number in the control condition to produce a relative ratio. The relative counts were then plotted for each set of method-specific sites. The x-axis is the relative distance of deletions to the peak centers and the y-axis is the relative counts.

dCLIP identified a total of 419 sites that were more enriched in the miR-124-transfected than the control cells, with an average tag count of at least five in the miR-124 transfected cells. We ranked target sites identified by Piranha by *P-*value and chose a cutoff that resulted in 418 final sites, in order to match the number of sites identified by dCLIP. There were a total of 202 common sites found by both methods. We then selected sites that could only be found by dCLIP (217) and those could only be found by Piranha (216) to conduct downstream comparison. First, we searched for motifs matching to any 7-mer from the reverse-complement of the miR-124 mature sequence within the RNA sequences of method-specific target sites. We plotted the motif matches relative to the target site centers in Figure 9a,b. The sequences of the dCLIP-specific sites contained 95 7-mer matches, of which 85% were within 20 bp of the target site centers. By comparison, the sequences of the Piranha-specific sites only contain 41 7-mer matches, of which 58% were within 20 bp of the target site centers. We also plotted the total motif matches found by each method in Figure 9c,d. Second, we investigated the number of deletions around peak centers. Since deletions are the characteristic mutations of RBP-binding sites in AGO HITS-CLIP experiments [8], we expected to find more deletions in the true differential binding sites. We counted the number of deletion mutations within the method-specific targets in both the miR-124 transfected and control cells. We divided the deletion counts in the miR-124-transfected cells by the mean number of deletions in the control cells and plotted the relative deletion counts for each method (Figure 9e,f). The dCLIP-specific targets provided a much higher relative count of deletion mutations than the Piranha-specific targets. In conclusion, the results of both motif matches to miR-124 and deletion mutation counts suggest that dCLIP was able to identify more biologically meaningful target sites than the Piranha software.

### TDP-43 iCLIP dataset

dCLIP is also able to analyze iCLIP datasets. The major difference of processing iCLIP datasets from HITS-CLIP and PAR-CLIP datasets is that cDNA counts, rather than total tag counts, are analyzed by the algorithm and no mutant tag counts are collected. We downloaded the TDP-43 iCLIP datasets from [41]. The TDP-43 RBP protein is mainly localized to the nucleus, and is involved in transcription, alternative splicing and the development of many diseases [42]. Aggregation of misfolded TDP-43 has been implicated in the neurodegenerative diseases frontotemporal lobar degeneration (FTLD) and amyotrophic lateral sclerosis [43]. In this study, the authors conducted iCLIP experiments with human postmortem cortical tissue from three healthy individuals and three patients who had sporadic FTLD with TDP-43 inclusions. The sequencing data from both sets of participants were pooled before mapping and we used an in-house program to remove PCR duplicates and trim the barcodes. We then mapped the sequencing tags to the hg19 reference genome and used dCLIP with a bin size of four nucleotides to analyze the alignment files. We also compared the performance of Piranha (PoissonRegression) with dCLIP on this iCLIP dataset.

**Figure 10:**
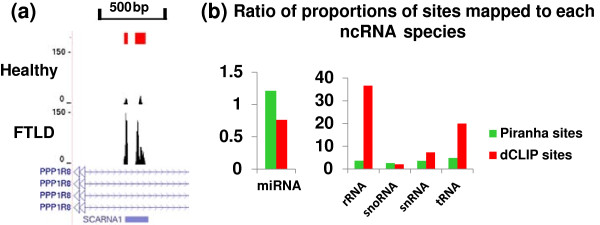
**The analysis results of dCLIP on the TDP-43 iCLIP datasets. (a)** An example of TDP-43 binding site on the ACA35 snoRNA with stronger binding in postmortem FTLD brains than healthy brains. The cDNA counts are shown. Red bars indicate regions with stronger binding in the FTLD brain. The height of each peak represents un-normalized cDNA counts. **(b)** Ratios of proportions of sites mapped to each ncRNA species. The ratio is calculated as the proportion of sites found to have stronger binding in the FTLD condition by dCLIP, mapped to each ncRNA species, divided by the proportion of sites having stronger binding in the healthy individuals mapped to the same ncRNA species. The ratios are also calculated for the differential binding sites found by Piranha, for comparison with dCLIP. FTLD, frontotemporal lobar degeneration; ncRNA, non-coding RNA, rRNA, ribosomal RNA; snoRNA, small nucleolar RNA; snRNA, small nuclear RNA; tRNA, transfer RNA.

The original publication determined that the FTLD iCLIP samples, compared to healthy samples, had increased binding of TDP-43 to small nucleolar RNAs (snoRNAs), small nuclear RNAs (snRNAs), transfer RNAs (tRNAs) and ribosomal RNAs (rRNAs), while binding to miRNAs decreased [41]. Figure 10a shows an example, in which the TDP-43 protein bound more strongly to the ACA35 snoRNA (SCARNA1) in the patients with FTLD than in healthy individuals. To examine this on a genome-wide scale, we calculated the proportion of sites with stronger binding in the FTLD tissues that could be mapped to each of the non-coding RNA species divided by the proportion of sites with stronger binding in the healthy condition that could be mapped to the same non-coding RNA species. Indeed, we confirmed the original publication’s finding by the fact that the sites found to have stronger binding in the FTLD brains by dCLIP are more likely to be mapped to snoRNAs, snRNAs, tRNAs and rRNAs and less likely to be mapped to miRNAs, as compared to sites with stronger binding in the healthy controls (Figure 10b). The ratios of proportions calculated from differential binding sites found by Piranha for snoRNAs, snRNAs, tRNAs and rRNAs were also >1; however, the ratio for miRNAs was approximately 1.2, inconsistent with the original publication’s finding. Moreover, this bias in annotation, reflected by the ratios of proportions, was more dramatic in differential binding sites found by dCLIP than in differential binding sites found by Piranha, for rRNAs, snRNAs and tRNAs (with only one exception for snoRNA). These results suggest that dCLIP is able to properly analyze iCLIP datasets and also performs better than Piranha.

## Discussion

The two-stage procedure implemented in dCLIP includes an MA normalization step and a HMM to identify differential and common binding sites. The MA normalization is a critical step to make the CLIP-seq data comparable across conditions. The straightforward rescaling by the total number of reads across samples is not appropriate for comparative CLIP-seq analysis because the signal-to-noise ratio usually varies across different conditions. The modified MA plot normalization method in dCLIP not only addresses the issue of different signal and noise levels effectively, but also works on much smaller units than those used for microarray and ChIP-seq data analysis, allowing dCLIP to detect binding sites of higher resolution required for CLIP-seq data analysis. To reduce potential bias and conduct rigorous comparison across different conditions, we recommend adopting the same experimental and bioinformatics procedures, such as RNase digestion, high-throughput sequencing and alignment, for both conditions.

The HMM plays a key role in identifying differential and common binding sites of two CLIP-seq samples in the dCLIP software. HMM can increase signal-to-noise ratios for sequencing data analysis, because it takes into account the correlation between consecutive bins. This is particularly important for CLIP-seq data, because of small bin size and high correlations between consecutive bins. The HMM in dCLIP defined a common binding state and two differential binding states. One thing to note for the three-state HMM is that the identified differential binding sites, for example the ones with inferred state of enriched and non-enriched, may actually only have a small tag enrichment in condition one, and an even smaller tag enrichment in condition two. Therefore, the differential binding sites need to be ranked and screened as such sites may not be of real interest to biologists. The analysis of the miR-155/AGO HITS-CLIP dataset, for example, set a cutoff of average tag intensity of 30 in the wild-type condition.

One assumption of the dCLIP algorithm is that most sites will not have changes in their binding between conditions. Our simulation studies (Additional file 4) show that dCLIP is able to handle comparative CLIP-seq analysis when there are more than 50% of common binding sites between two samples. We recommend users to roughly assess whether this assumption is valid or not for their experiments based on biological knowledge or preliminary bioinformatics analysis. In addition, dCLIP software will issue a warning if the estimated proportion of common binding sites with similar binding strength is less than 50%.

The dCLIP software was benchmarked against the Piranha software. Piranha incorporates covariates which could represent transcript abundance, count data in the second condition or positional mutation information. However, the covariate is incorporated in the statistical model in the exactly same way no matter which type of data it actually represents. This design enables Piranha to be easily applied to a wide variety of CLIP-seq data analysis scenarios. However, this one-for-all method also harms the detection power of RBP binding regions of interest in each specific scenario, as different data types have their unique properties and should be treated differently. The dCLIP method is specialized in comparing two CLIP-seq experiments and was shown to perform better than Piranha in identifying differential binding sites. Therefore dCLIP should be a better choice when the users are interested in identifying differential or common RBP-binding sites.

The pairwise approach to compare CLIP-seq data in dCLIP can be extended to multiple-sample comparison. When there are n samples, a transition matrix of 2^*n*^ states need to be implemented in the HMM. Theoretically, dCLIP can be easily modified to handle as many samples as possible. However, if n exceeds 10, the computation cost will increase dramatically. In addition, the normalization method also needs to be changed to suit the multiple-sample comparison. For example, the trimmed mean of M values method [44] or the upper-quartile normalization method [45] could be modified to handle the normalization step for multiple-sample comparisons. Currently, most CLIP-seq studies do not conduct transcript abundance measurements [29,46] and, accordingly, most current CLIP-seq analysis tools, such as PARalyzer [13], do not consider transcript abundance either. However, taking background transcript abundance into account will be very helpful for more accurately defining RBP binding sites in either one-sample scenarios or multiple-sample scenarios. If the background expression data is available, that information can be relatively easily incorporated into dCLIP to further refine its performance.

We present a new computational approach, dCLIP, for the comparative analysis of CLIP-seq data. dCLIP was implemented as an easy-to-use command line tool in the Perl programming language. The dCLIP software is able to handle HITS-CLIP, PAR-CLIP and iCLIP datasets, and can take single-end or paired-end sequencing files as input. The dCLIP software is strand-sensitive and is able to detect differential binding sites at almost single-base resolution. It also correctly keeps all of the characteristic mutation information for later analysis. Real data analysis shows that dCLIP can accurately identify differential binding regions of RBPs and outperforms another CLIP analysis program, Piranha [16]. We anticipate that the dCLIP software will become a helpful tool for biologists and bioinformaticians for comparative CLIP-seq data analysis.

## Abbreviations

AGO: argonaute; bp: base pair; ChIP: chromatin immunoprecipitation; CLIP-seq: crosslinking immunoprecipitation coupled with high-throughput sequencing; FTLD: frontotemporal lobar degeneration; HITS-CLIP: high-throughput sequencing of RNA isolated by crosslinking immunoprecipitation; HMM: hidden Markov model; iCLIP: individual-nucleotide resolution crosslinking and immunoprecipitation; miRNA/miR: microRNA; PAR-CLIP: photoactivatable-ribonucleoside-enhanced crosslinking and immunoprecipitation; PCR: polymerase chain reaction; phyloP: phylogenetic p-score; RBP: RNA-binding protein; rRNA: ribosomal RNA; snoRNA: small nucleolar RNA; snRNA: small nuclear RNA; tRNA: transfer RNA; UCSC: University of California Santa Cruz; UTR: untranslated region; RIP-seq: RNP immunoprecipitation followed by high-throughput sequencing.

## Competing interests

The authors declare that they have no competing interests.

## Authors’ contributions

TW, YX and GX designed the project and developed the underlying algorithms. TW designed and developed the software, performed all of the testing and analysis and wrote the user guide. All authors wrote, read and approved the final manuscript.

## Supplementary Material

Additional file 1 Source code of the dCLIP software 1.0. Click here for file

Additional file 2 User guide for the dCLIP software 1.0. Click here for file

Additional file 3 Exemplary output files of the dCLIP software. Click here for file

Additional file 4 Simulation results to test the robustness of the dCLIP software. Click here for file
