# Supplementary material for: dCLIP: a computational approach for comparative CLIP-seq analyses
Source: Genome Biol. 2014 Jan 7;15(1):R11. doi: 10.1186/gb-2014-15-1-r11 (PMC4054096; doi:10.1186/gb-2014-15-1-r11)
Supplement: Additional file 2 — User guide for the dCLIP software 1.0. [file gb-2014-15-1-r11-S2.pdf]

==
==================== User's Guide for dCLIP =====================
==

WHAT IS dCLIP

dCLIP is written in Perl for discovering differential binding regions in two CLIP-Seq (HITS-CLIP or PAR-CLIP) experiments. It is appropiate in experiments where the common binding regions that are significantly enriched in both conditions tend to have similar binding strength and when researchers are more interested in the difference in binding strength rather than the binary event of whether binding site is common or not. For example, dCLIP will work when researchers would like to know the differential binding sites of AGO protein under a wild-type and miRNA knockdown condition.

In dCLIP analysis, duplicate reads that have the same mapping coordinates are collapsed and only one read with the highest number of mutations is retained. Then dCLIP identifies CLIP clusters from the remaining reads of both experiments. Then dCLIP would divide CLIP clusters into bins of the same size, count tag intensities in each bin and normalizes the two conditions by MA plot followed by linear regression. Using the normalized M values, dCLIP implements a Hidden Markov Model and Viterbi algorithm to identify differential and common binding regions.

The CLIP-Seq experiments can be done in single-end mode or paired-end mode but must be in a strand-specific manner. dCLIP takes the SAM format files of two experiments as input and produces 10 files as output. Most of the output files can be uploaded to UCSC Genome Browser for visualization. dCLIP only uses tag enrichment for inference of differential binding regions. Mutation information is not used for statistical inference but is still collected and written to output files. In addition, the users can choose a specific mutation type or combination of mutations types as characteristic mutations. For example, "T2C" for PAR-CLIP and "Del" for HITS-CLIP.

INSTALLATION

You should have a perl version >= 5.16 to run dCLIP. Also you need to install Perl's PDL module and PDL::Stats module to run dCLIP. If you don't have or don't know how to install this package. Please check the MODULE file, which has some suggestions for installing Perl modules. With it installed, just use the following command to decompress the package

```
$ tar zxvf dCLIP_0.1.tar.gz
```

And the dCLIP.pl script will be placed in the bin directory, you can add dCLIP bin folder to your path. To call the software, use

```
$ perl dCLIP.pl [options]
```

RUNNING dCLIP

Input:

-f1 The SAM format file of the first condition.

-f2 The SAM format file of the second condition.

-pair If the aligned SAM format files are from single-end experiments, leave this option unset. For paired-end files, set this option to the suffix of the names of forward reads and backward reads. For example, "F3,F5-RNA".

-m1 The minimum number of tags for the first condition. All tags from both conditions are pooled, collapsed and overlapped to form clusters. Only clusters with at least m1 tags of the first condition or m2 tags of the second condition will be considered. Default: 5.

-m2 The minimum number of tags for the second condition. Default: 5.

Directory:

-temp The temporary directory to store intermediate files. Default: ".".

-dir The folder to store final output files. Default: ".".

Parameters:

-step The step size of profiling tag intensities. This controls the resolution of the Hidden Markov Model. Default: 5.

-filter A filter value used for defining regions with significant binding in both conditions. A higher value will be more conservative in calling differential regions. Should be set >1. Default: 10.

-mut The mutant type(s) of the marker mutations. Can be any one or combination (separated by comma) of "T2C","T2A",...,"A2G","Del","Ins". For example, "T2C,A2G" will include T-to-C and A-to-G mutations as marker mutations. "all" will include all types of mutations. Default: "T2C".

-max The maximum number of iterations allowed for the Hidden Markov Model. Default: 10.

-pre The precision of the criterion for convergence. Default: 0.001.

Help:

-h Print this help message.

MORE DETAILS

1) For paired-end experiments, reads are usually named with suffix specifying the forward and backward segment for the same read. For example,

```
604_829_626_F5-RNA
604_829_626_F3
167_570_1179_F3
167_570_1179_F5-RNA
```

"F3" means forward strand and "F5-RNA" means backward strand, so the pair parameter should be set to F3,F5-RNA in this case. In other cases, the pair paramter may be set to 1,2 or other character strings. Sometimes, the aligner will trim the suffix. For example, "HWI-

ST188:8:2217:5190:132924\#0/1" and "HWI-ST188:8:2217:5190:132924\#0/2" are one mate and certain aligners will only write "HWI-ST188:8:2207:5196:132923\#0" for both segments in the alignment file. In such cases, please set the suffix to "" and "" or "\#0" and "\#0". The point is to make the remaining part of the read names the same for a mate.

2) dCLIP treats paired-end sequencing data as fr-secondstrand by default (consult Tophat manul for definition). It can also handle fr-firststrand if you use opposite bases when specifying substitution mutations. For example, instead of specify T2C, you can use A2G. dCLIP cannot handle any other type of fr or any type of ff libraries.

2) The m1 and m2 parameters are designed to limit differential analysis to larger clusters. For those small clusters with only a few reads, they are not very interesting for biologists not matter whether they have differential binding strength or not. Also, bigger m1 and m2 values will make the program run faster. I would recommend a value between 5-15 for m1 and m2.

3) For PAR-CLIP experiments, the characteristic mutation type (mut) is "T2C" or "G2A" depending on the nucleoside analog used. For HITS-CLIP experiments, it has been suggested that "Del" mutation is characteristic of AGO and Nova proteins. However, this may not be the case for other proteins. So if the user is interested in mutation information, it may be a good idea to try different types and combinations of mutations and to see which setting makes the most sense from downstream analysis.

4) Reads with gapped alignments are discarded. However, gapped-aligned reads usually comprise a very small portion of total reads (<2%) so it should be ok to neglect these alginments.

5) For one experimental condition with more than one replicate, it is ok to simply concatenate the SAM format alignment files like this

```
$ cat file1.sam file2.sam file3.sam > file.sam
```

OUTPUT FORMAT
=============

The program will produce 10 output files in the output folder.

1) 8 of these files named like "File1_Mutant_neg.bedgraph" are bedGraph format files storing the total or mutant tag counts on the + or - strand in the first or second condition. For example, "File1_Mutant_neg.bedgraph" stores the mutant tag count on the - strand in the first condition as a bedGraph format file. The resolution of these files is 1bp. These files can be uploaded to UCSC Genome Browser for visualization.

2) "dCLIP_output.txt" stores the detailed information of the raw data and Hidden Markov Model inference results at the resolution of the step bp. The first id column is an id number of the CLIP clusters. The chrom, strand and position specify the genomic location of each bin of step size. The next two

columns, state and probability, specify the inference results of the Hidden Markov Model. The differential column is the normalized difference of the tag intensities between the two conditions. The last four column, tag1, mut1, tag2 and mut2, specify the total tag intensity and mutatant tag number of the two conditions in each bin.

Note: For the state column,
0 refers to a bin with more binding in condition 2 than condition 1
1 refers to a bin with equal binding in both conditions
2 refers to a bin with more binding in condition 1 than condition 2

3) "dCLIP_summary.bed" stores the summary of the inference results. Neighbouring bins in the same cluster with the same inference results are collapsed to be represented as one region in the BED file. The fourth column is the same id number as used in the dCLIP_output.txt file. The fifth column is the average binding strength of condition 1 in this continuous region if in state 2, the average binding strength of condition 2 if in state 0 and 0 if in state 1. I recommend to use this value for ranking regions with differential binding (state 0 and state 2 regions). This file can be uploaded to UCSC Genome Browser.

Note: For the color coding
Red bars are regions where condition 2 has stronger binding than condition 1
Green bars are regions with equal binding strength for both conditions
Blue bars are regions where condition 1 has stronger binding than condition 2

EXEMPLARY DATA

In the test folder there are two demo SAM files for demonstrating the usage of the dCLIP software. These two files are a small portion of the published dataset GSE41285. Here is a link to the original publication: http://www.sciencedirect.com/science/article/pii/S1097276512008544. Please cd into the dCLIP directory and run the following command. Results will also be placed in the test folder.

```
$ perl bin/dCLIP.pl -f1 test/knockdown.sam -f2 test/wildtype.sam -m1 2 -m2 2 -temp test -dir test -mut "T2C"
```

OTHER

Version: 1.0
Author: Tao Wang
Email: tao.wang@utsouthwestern.edu
