# Supplementary material for: dCLIP: a computational approach for comparative CLIP-seq analyses
Source: Genome Biol. 2014 Jan 7;15(1):R11. doi: 10.1186/gb-2014-15-1-r11 (PMC4054096; doi:10.1186/gb-2014-15-1-r11)
Supplement: Additional file 3 — Exemplary output files of the dCLIP software. [file gb-2014-15-1-r11-S3.pdf]

```
$ head dCLIP_summary.bed
track name="dCLIP_summary" description="dCLIP_summary" itemRgb="On"

chr18  3383080  3383179      1      0  +      3383080  3383179  0,255,0
chr18  3383280  3383419      2      0  +      3383280  3383419  0,255,0
chr18  3399830  3399839      3      0  +      3399830  3399839  0,255,0
chr18  3399840  3399889      3     22  +      3399840  3399889  0,0,255
chr18  3399890  3399909      3      0  +      3399890  3399909  0,255,0
chr18  3399910  3399969      3     25  +      3399910  3399969  255,0,0
chr18  3399970  3399999      3      0  +      3399970  3399999  0,255,0
chr18  3405680  3405719      4     22  +      3405680  3405719  0,0,255
chr18  3405720  3405789      4      0  +      3405720  3405789  0,255,0
```

```
$ head dCLIP_output.txt

id  chrom   strand  position  state  probability  differential  tag1  mut1  tag2  mut2
 1  chr18   +        3383080      1        1.000         0.104     7     0     0     0
 1  chr18   +        3383090      1        0.996         0.180    16     0     0     0
 1  chr18   +        3383100      1        0.989         0.212    20     0     0     0
 1  chr18   +        3383110      1        0.977         0.235    23     0     0     0
 1  chr18   +        3383120      1        0.963         0.265    27     0     0     0
 1  chr18   +        3383130      1        0.955         0.280    29     0     0     0
 1  chr18   +        3383140      1        0.960         0.277    33     0     3     0
 1  chr18   +        3383150      1        0.977         0.228    50     0    20     0
 1  chr18   +        3383160      1        0.988         0.221    49     0    20     0
```

```
$ head File1_Mutant_pos.bedgraph
track type=bedGraph name="File1_Mutant_pos" description="File1_Mutant_pos"

chr18  3399865  3399866      2
chr18  3414156  3414157      2
chr18  3417607  3417608      1
chr18  3421257  3421258      1
chr18  3431526  3431527      1
chr18  3431610  3431611      1
chr18  3434051  3434052      3
chr18  3435081  3435082      1
chr18  3435322  3435323      1
```
