# Supplementary material for: dCLIP: a computational approach for comparative CLIP-seq analyses
Source: Genome Biol. 2014 Jan 7;15(1):R11. doi: 10.1186/gb-2014-15-1-r11 (PMC4054096; doi:10.1186/gb-2014-15-1-r11)
Supplement: Additional file 4 — Simulation results to test the robustness of the dCLIP software. [file gb-2014-15-1-r11-S4.docx]

One assumption of dCLIP algorithm is that most sites will not have changes in their binding between conditions. To test the robustness of dCLIP on this assumption, we carried out simulation studies, in which we assumed different proportions of common binding sites and investigated the performance of dCLIP. To be more specific, we assumed that $\mu=2,\sigma=0.5$and simulated 30,000 bins on the same chromosome. Every segment of 30 adjacent bins was assumed to be in the same state, corresponding to the spatial dependencies between bins, while each segment was assumed to take the state of 0, 1 and 2 with probability of (0.5-p/2),p and (0.5-p/2). Then setting p to 0.7, for example, will render 70% of all bins to be common protein-binding regions. Given the “true” status of each bin, an adjusted M value that follows a normal distribution can be sampled and the corresponding tag counts for both conditions can be sampled.

We then ran dCLIP on the simulated data with varying values of p. dCLIP gives the inferred state for each bin and an associated probability for each bin in the inferred state. For each p, we treat the probabilities as the prediction scores and the Boolean value of whether the inferred state is the same as the true state as the binary label. Therefore, we could draw an ROC plot showing the false positive rate and true positive rate of inference of all 30,000 bins for each p (proportion of common binding sites). From **RL. Fig. 6**, we can see that even if the true proportion of common binding sites is only 50%, dCLIP could still give reasonable results. If the proportion of common binding sites increases to 70%, we can observe a huge improvement in the performance of dCLIP. When there are 90% of true common binding regions, the dCLIP software has an almost 0 false positive rate in identifying the hidden state of each bin. However, when there are less than 50% of common binding regions, the performance of dCLIP is not ideal.

In conclusion, the simulation results here suggest that dCLIP is able to handle comparative CLIP-Seq analysis when there are more than 50% of common binding sites (a likely reasonable presumption). It will be helpful if the user can judge from the biological point of whether this assumption is met for the experimental system or if the user can conduct some preliminary bioinformatics analysis to roughly assess the validity of this assumption. In addition, the dCLIP algorithm will automatically issue a warning if the fitted p suggests that the proportion of common binding sites with similar binding strength is less than 50%.


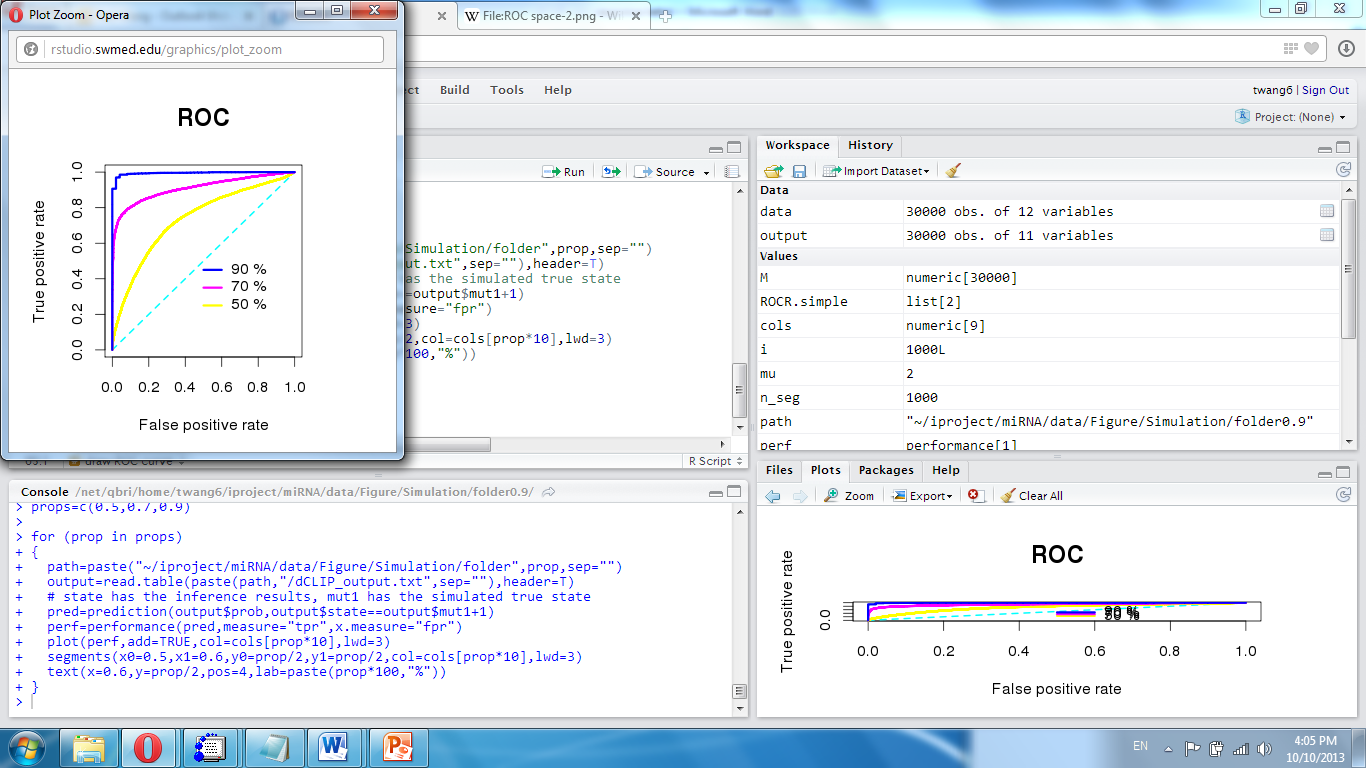


The ROC curve of dCLIP’s prediction accuracy when the true proportion of common binding sites with similar binding strength is 90%, 70% and 50%.
